# A Bayesian approach for applying Haseman-Elston methods

**DOI:** 10.1186/1471-2156-6-S1-S39

**Published:** 2005-12-30

**Authors:** Seungtai Yoon, Young Ju Suh, Nancy Role Mendell, Kenny Qian Ye

**Affiliations:** 1 Department of Applied Mathematics and Statistics, State University of New York at Stony Brook, Stony Brook, NY 11794, USA; 2 The Institute of Natural Sciences, Sookmyung Women's University, Seoul, Korea; 3 Albert Einstein College of Medicine, Bronx, NY 10461, USA

## Abstract

The main goal of this paper is to couple the Haseman-Elston method with a simple yet effective Bayesian factor-screening approach. This approach selects markers by considering a set of multigenic models that include epistasis effects. The markers are ranked based on their marginal posterior probability. A significant improvement over our previously proposed Bayesian variable selection methodology is a simple Metropolis-Hasting algorithm that requires minimum tuning on the prior settings. The algorithm, however, is also flexible enough for us to easily incorporate our hypotheses and avoid computational pitfalls. We apply our approach to the microsatellite data of Collaborative Studies on Genetics of Alcoholism using the coded values for the ALDX1 variable as our response.

## Background

The Haseman-Elston method is an effective method for studying linkage between markers and diseases. Given the genotypes and phenotypes of a set of sib-pairs, it regresses a statistic measuring similarity of quantitative traits in the sibling pair on the number of alleles identical by descent (IBD) at each marker. The original Haseman-Elston methods used the squared difference between the trait values of the siblings as the measure of similarity. More recently, Elston et al. [1] proposed the cross-product of the trait values as the response. Several recent papers suggested other statistics as the response. Feingold [2] gave a comprehensive review of these regression-based linkage methods.

The simple regression setup of the Haseman-Elston method allows one to easily study simultaneous effects of several loci and epistasis effects. It also allows one to apply model selection methods developed for regression analysis. In the Genetic Analysis Workshop 12, Suh et al. [3] explored these possibilities by applying stochastic search variable selection (SSVS), a Bayesian variable selection method proposed by George and McCulloch [4] for linear regression models, to the Haseman-Elston method. In the Genetic Analysis Workshop 13, Oh et al. [5] took a further step to consider 399 markers as well as their interactions using the same SSVS algorithm. In addition, they proposed using the marginal posterior of each variable, both main effects and interaction effects, to rank their importance. A known deficiency of SSVS is that its results are sensitive to specification of a number of parameters in prior settings. The effects of the choice of different prior settings are also not well understood. Oh et al. [5] also adopted a method developed by Chipman [6] to impose a hierarchical prior structure on the model space. Such a hierarchical prior reflects an *a priori *belief in certain types of models but further complicates the algorithm and adds computation burdens.

The goal of this work is to propose a simple and effective Bayesian variable selection method that requires minimum specification of prior settings and leaves flexibility to impose hypotheses on the model space. Another notable difference in our new approach from our previous approaches [3,5] is that we make inference only on the markers but not specific interaction effects. However, when we evaluate a marker, its interactive effects with other markers are considered in additional to its main effects. The goal of our approach is to identify markers that are potentially related to the phenotype but not to find a genetic model that explains the phenotype.

## Methods

We chose to analyze ALDX1 measured in the Collaborative Study on the Genetics of Alcoholism study, using microsatellite genotype information. We only took those individuals classified in ALDX1 as "purely unaffected", coded 1, "unaffected with some symptoms", coded 3, "affected", and coded 5. Among those individuals, we used GENEHUNTER to obtain IBD values for all sib-pairs. We used the cross product *CP_i _*= (*Y*_1*i *_- *m*)(*Y*_2*i *_- *m*) as our measure of similarity of phenotype in the pair. Here *m *is the mean of all individuals in the sib group. We also used squared difference  for comparison. We also included the sex as an explanatory variable. Sex was coded "0" if the sib pair was of the same gender and "1" if not. For simplicity, we assumed the errors to be independent and normally distributed, but the correlation structure could be implemented into our method in a straightforward way. Therefore, without loss of generality, we have



where *ε*~N(0, *σ*^2^) are assumed to be independent, and *X*_*j *_values are either IBD values at markers, or measures of similarity for some covariate. We refer to these marker loci and covariates as *factors *in our discussion. The above model includes main effects as well as two-factor interaction effects and three-way interaction effects but can be extended to include higher order interaction effects.

Oh et al. [5] used SSVS to obtain a Markov chain Monte Carlo (MCMC) sample from the posterior of the model space of *2*^*p *^models, where *p *is the total number of effects (i.e., main effects, epistasis effects) in the above equation. Each model corresponds to a subset of *p *effects being active. The marginal posterior of each effect is then obtained to evaluate the importance of the effect. When interaction effects are considered, the model space becomes extremely large so that the MCMC algorithm is less effective. Therefore, we propose an approach that reduces the model size dramatically while considering the interaction effects. Our model space only contains  models, where *m *is the total number of factors (genetic markers and/or environment factors), *often much smaller than p*, and *l *is the number of factors involved in a model. The choice of *l *reflects the number of factors believed to be associated with the disease. Each model in our model space corresponds one-to-one to a subset of *l *elements from *n *marker loci and covariates. *Each model contains the main effects of l factors and interaction effects up to a certain order among those factors*. The effects involving other factors are not included in the model, i.e., their corresponding coefficients in Equation (1) are set to be zero. Because all models in our model space have the same number of parameters, they are assigned the same prior probability. A model in our model space can be represented by a binary vector ***γ ***= (*γ*_1_, *γ*_2_, ..., *γ*_*n*_), where *γ*_*i *_= 1 indicates the *i*^th ^factor being active and . For a model *γ*, we denote the active coefficients as *β*_*γ *_in which the first element always corresponds to the constant term, and the model matrix as *X*_*γ*_. The prior of *β*_*γ *_is given as *N*(0, *σ*^2^Γ^-1^) where . The variance of the error term is assigned a non-informative prior *f*(*σ*) ∞ 1 / *σ*. By integrating out *β*_*γ*_, the posterior of model *γ *can be shown to be



We adopt the Metropolis-Hasting algorithm to sample from the model space and obtain a sequence *γ*^(0)^, *γ*^(1)^, ..., *γ*^(*j*)^, .... At the (*j *+ 1) th step, a model γ* is selected by replacing a random active factor in *γ*^(*j*) ^by an random inactive factor not in *γ*^(*j*)^. Set *γ*^(*j *+ 1) ^= γ* with probability min {*f*(γ*|*y*) / *f*(*γ*^(*j *+ 1) ^| *y*),1}; otherwise, *γ*^(*j *+ 1) ^= *γ*^(*j*)^. The marginal posterior of all factors are estimated from the MCMC sample to measure the importance of those factors. It is important to note here that the posterior probability of a factor not only captures its main effects but also its interaction effects with other factors.

Our study is conducted with two settings, one with square of difference, D^2^, and the other with cross product, CP. There were 224 nuclear families and 1,499 sib pairs. The mean in CP was the average of each family. There are total of 315 candidate markers treated as factors. An additional factor is sex.

We set *l *= 3, 4, 5 to reflect our belief that no more than 5 factors have a significant contribution. We consider models with main effects and all two-factor interactions. Each model in the model space has intercept, main effects and all two-factor interaction effects. The total number of parameters in the models are 7, 11, and 16 respectively for l = 3, 4, 5. We ran the Metroplis-Hasting Markov Chains 100,000 iterations for 3, 4-factor models and 500,000 for 5-factor model. To check for convergence, we ran two chains of 100,000 with different random number generating seeds for each case. For a prior of *β*_*γ*_, we chose *λ *= 1.5 in our computation. This reflects a belief that the average size of *β*_*γ *_is 1.5 times of the error term. This choice follows the recommendation of Box and Meyer [7]. In addition, we also find the choice of *λ *in a greater range, 1 <*λ *< 15 does not affect the results much. We illustrated this robustness in Figure 3, in which marginal probability plots from *λ *= 1.5 and *λ *= 10 are displayed side by side. The two plots show identical main features.

## Results and Discussion

The methods described in the previous section were implemented using JAVA programming language for its portability. The program runs successfully under Linux and Mac OS X operating systems.

**Figure 1:**
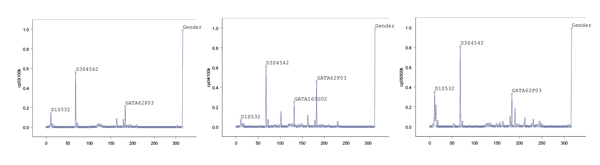
**Marginal probability plots with CP as the response. **Marginal frequency plots of markers with CP as the response. The right shows the result of *l *= 3. The middle shows the results of *l *= 4, and the left shows the results of *l *= 5. The markers are ordered by their positions on the genome. The 316^th ^factor is sex (gender).

**Figure 2:**
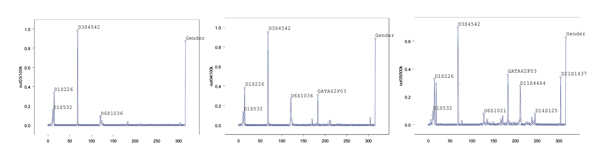
**Marginal probability plots with D^2 ^as the response. **Marginal frequency plots with D^2 ^as the response. The left shows the result of *l *= 3. The middle shows the results of *l *= 4, and the right shows the results of *l *= 5. The markers are ordered by their positions on the genome. The 316^th ^factor is sex (gender).

**Figure 3:**
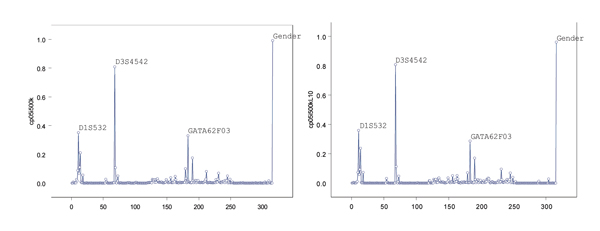
**Marginal probability plots with different *λ *values. **Marginal frequency plots of markers with CP as the response. The left shows the result of *λ *= 1.5. The right shows the results of *λ *= 10. These plots show that the choice of *λ *has no significant effect on the output.

Figure 1 shows marginal frequency plots with CP as the response and Figure 2 shows the result with D^2 ^as the response. It can be easily seen from the plots that the results using CP and D^2 ^are very similar. Both show that D3S4542 and sex have very high marginal posterior probabilities. This suggests that marker D3S4542 and sex are strongly associated with ALDX1.

As model size increases, the overall frequency was higher, as we expected. Factors with high frequency are amplified and new factors with higher frequency appear. Marker GATA62F03 in chromosome 9 shows relatively high marginal probability in most of the plots, as does marker D1S226 on chromosome 1. These two markers also show evidence of linkage with ALDX1, but not as strong as D3S4542 and sex. We see an interesting region around the D6S493. For D^2^, the plot shows some spikes and for CP, the plot shows relatively wide discontinuity in this area. It could be due to strong correlation between the IBD values or something associated with ADXL1. We would need to do further investigation of this region to address this issue.

Although one can visually inspect those plots and determine which markers are important, more formal statistical inferences are desired. However, this topic is not in the scope of this paper and will be addressed in the future.

## Conclusion

In this paper, we applied a Bayesian method to select important markers using Haseman-Elston methods. The likelihood of a marker associated with a phenotype is evaluated by a posterior probability that captures not only main effects but also all interaction effects of the marker. The method is much simpler than previously proposed Bayesian variable selection methods and does not require fine tuning the prior settings. The result also shows the consistency and robustness of our method. Our approach is similar to the Bayesian factor screening method proposed by Box and Meyer [7]. A major difference between our approach and theirs is that we only consider models with same number of active factors, and they consider all 2^*m *^models. An important advantage of our approach is that we can assign equal priors to all models in our model space. Assigning priors to models of different sizes has many complications as discussed by Chipman et al. [8] in great detail.

## Abbreviations

IBD: Identical by descent

MCMC: Markov chain Monte Carlo

SSVS: Stochastic search variable selection

## Authors' contributions

KQY led the effort of this work. SY carried out the algorithmic development as well as the computational effort. NRM and YJS both contributed to the main ideas of this paper.
